# Dynamic deformation of femur during medial compartment knee osteoarthritis

**DOI:** 10.1371/journal.pone.0226795

**Published:** 2019-12-20

**Authors:** Yang Lu, Zhanle Zheng, Wei Chen, Hongzhi Lv, Ji Lv, Yingze Zhang

**Affiliations:** 1 Department of Emergency Surgery, The First Hospital of Qinhuangdao Affiliated to Hebei Medical University, Haigang District, Qinhuangdao, Hebei Province, People’s Republic of China; 2 Department of Orthopedic Surgery, The Third Hospital of Hebei Medical University, Qiaoxi District, Shijiazhuang, People’s Republic of China; Mayo Clinic Minnesota, UNITED STATES

## Abstract

**Objectives:**

The aim of this study was to evaluate the morphological changes of the femur in the coronal plane in progressing varus gonarthrosis and to explore the interrelation of each component.

**Patients and methods:**

From January to July 2017, radiographic images of 1538 knees of 883 consecutive patients were collected and analyzed. We drew the alignments and measured the orientation angles of the lower extremities and compared the results among age groups for each sex. Correlation and regression tests were used to analyze the measurements.

**Results:**

There were significant differences in the neck-shaft angle (NSA), femoral bowing angle (FBA) and anatomic medial distal femoral angle (aMDFA) by age group in females, whereas the differences were not significant in males. In females, a positive correlation was found between age and the FBA and aMDFA (r = 0.253, 0.141, p<0.01), and a negative correlation was found between age and the NSA while the FBA was controlled (r = -0.065, p<0.05). The FBA was positively correlated with the NSA (r = 0.312, p<0.01) and aMDFA (r = 0.233, p<0.01). The NSA, FBA, and aMDFA together affected 72.2% of the mechanical medial distal femoral angle (mMDFA) (β = 0.071, -0.528, 0.803, p<0.01).

**Conclusion:**

As knee osteoarthritis (KOA) progressed, dynamic deformation of the femur was found in females, while no obvious changes were found in males. Femoral mechanical axis varus (mMDFA decrease) was the result of changes in the NSA, FBA and aMDFA. The deformation was throughout the femur rather than in a local area, as femur bowing can lead to corresponding changes in both ends of the femur. We provided a theoretical basis for TKA and knee-salvage treatment, and more attention should be paid to aging patients, especially females, in the preoperative protocol for orthomorphia.

## Introduction

The pathogenesis of knee osteoarthritis (KOA), which threatens the health of middle-aged and elderly individuals, includes biomechanical changes, inflammation, strain and degeneration; medial compartment KOA accompanied by knee varus is the most common type [1,2]. Medial tibial plateau collapse was considered the fusing of knee varus [3]. However, studies have indicated that femoral mechanical axis varus, which was seldom noticed, affected the hip-knee-ankle angle (HKA) almost the same as the tibial deformity in varus knees [4]. Our study focused on the femoral morphological changes inside of the femoral mechanical axis varus and aimed to investigate the possible correlations between them. The results could provide the data to support a theoretical basis for the etiology of knee arthritis and could be utilized as the data to support the use of total knee arthroplasty (TKA) and femoral osteotomy in knee-salvage treatment. Changes in the femoral mechanical axis are the key parts of the phenomenon of dynamic bone deformation throughout the body.

## Materials and methods

### Patients

The study was a retrospective review of patient records in accordance with the ethical standards of the Ethical Board Review of the Third Hospital of Hebei Medical University (Shijiazhuang, China) and with the Helsinki Declaration of 1975, as revised in 2000. We began to access the films after getting the approval of the Ethics Committee instead of before taking the films. The films were taken according to the patient’s medical needs, not for our research. We just read the X-ray films. Thus, the date we obtained ethical approval was after the filming date. All data were fully anonymized before we accessed them. Long-standing AP image-splicing radiographs of the lower extremities of consecutive patients who visited the orthopedic clinics in our hospital between January 2017 and July 2017 were collected and analyzed. Non-traumatic knee pain was the most common reason they went to the hospital. Some of them had obvious varus deformity of the knee joints with or without concomitant knee activity limitations. The standardized AP standing view was defined as the slight overlap of the proximal tibiofibular joint, accounting for approximately one third of the fibulae capitulum [5]. Congenital lower limb deformities, valgus knees, osteonecrosis of the femoral head, prior fractures or previous surgery of the lower limb, rheumatoid arthritis, ankylosing spondylitis, acute gout flaring, and metabolic bone diseases were excluded from this study. Additionally, inappropriate orthoroentgenograms (such as those taken without weight bearing, those with the knees in significant flexion and rotation, or those in which ankle or hip joints were obscure) were also eliminated. Long-standing AP image-splicing radiographs of the entire lower extremities included the complete hip, knee, and ankle joints. All radiographs were taken by two trained radiology technicians. The radiographs were obtained with the help of picture archiving and communication system (PACS) (Beijing Tianjianyuanda Technology Co., Ltd., Beijing, China). Radiographic assessments were performed using PACS and Digimizer image processing and graphical analysis software (MedCalc Software bvba, Ostend, Belgium, version 4.2.6.0). All radiographic measures were taken by the same observer (L.Y.) three times, and the mean value was used. All distances and angles were measured using calipers and goniometers provided by the PACS system.

In total, 1538 lower extremities of 883 patients were selected for the study, including 1187 limbs of 684 women and 199 limbs of 351 men, with a mean age (and standard deviation) of 60.86 ± 8.62 years (range, 17–87 years). The knees without varus or valgus (HKA = 180°) were also involved to better reflect the process of dynamic deformation.

### Radiographic assessment

As there is lack of a general agreement on nomenclature for lower extremity radiologic parameters, the radiographic assessment was partly made as described by Paley [6], mostly using his nomenclature; the remaining nomenclature was based on the nomenclature in common use. Paley’s principle was used for naming the mechanical medial distal femoral angle (mMDFA) and anatomic medial distal femoral angle (aMDFA). The difference is that Paley used the supplement angle instead. In contrast, we highlight the concept of the mMDFA and aMDFA for theoretical explanations of this study because they are located on the medial side of the knee joint, which bears the greater load.

**Fig 1:**
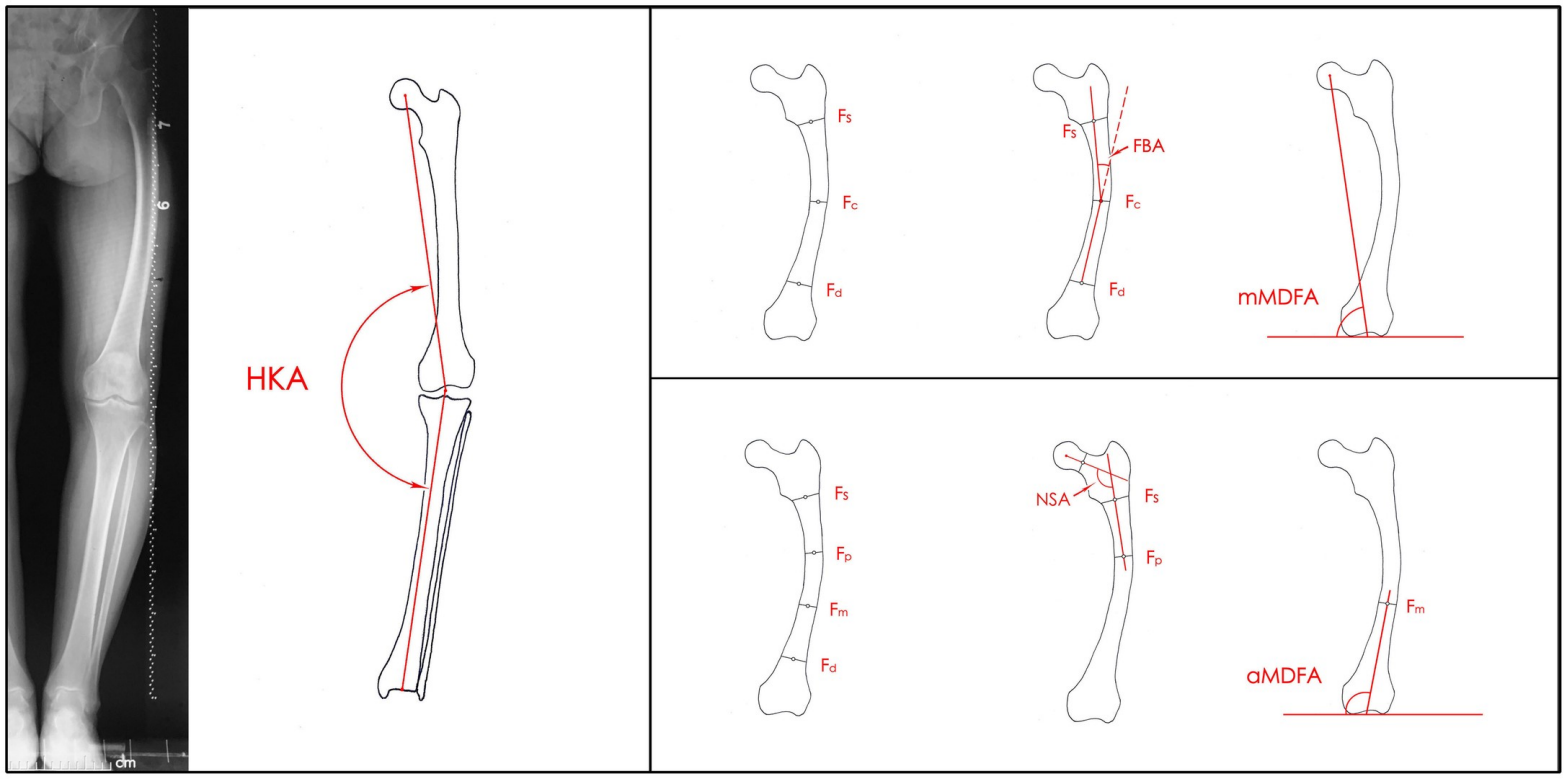
Radiographic measurements. **HKA**: hip-knee-ankle angle; **Fs**: a point bisecting the width of the femoral shaft at the lower junction of the lesser trochanter and the shaft; **Fd**: a point bisecting the width of the shaft 10 cm proximal to the knee joint; **Fp**: a point bisecting the width of the shaft at junction of the proximal third and the midsection; **Fm**: a point bisecting the width of the shaft at junction of the midsection and the distal third; **Fc**: a point bisecting the width of the femoral shaft midway between Fs and Fd; **NSA**: neck-shaft angle; **FBA**: femoral bowing angle; **mMDFA**: mechanical medial distal femoral angle; **aMDFA**: anatomic medial distal femoral angle.

Point **Fs** was a point bisecting the width of the femoral shaft at the lower junction of the lesser trochanter and the shaft. Point **Fd** was a point bisecting the width of the shaft 10 cm proximal to the knee joint. The femoral shaft was defined as the bone between **Fs** and **Fd**. Then, the length of the femoral shaft was trisected, and **Fp** was the point bisecting the width of the shaft at the junction of the proximal third and the midsection. The point bisecting the width of the shaft at the junction of the midsection and the distal third is shown as **Fm**. **Fc** was a point bisecting the width of the femoral shaft midway between **Fs** and **Fd**. The proximal femoral anatomical axis was a line connecting **Fs** and **Fp**. The distal femoral anatomical axis was a line connecting **Fm** to the center of the knee. **HKA** was the medial angle formed between the femoral mechanical axis and the tibial mechanical axis (varus, +; valgus, -) [7,8]. **mMDFA** was the medial angle formed between the mechanical axis line of the femur and the knee joint line of the femur in the frontal plane. The normal value of the mMDFA = 92° (from 90° to 95°). Femoral mechanical axis varus was defined as an mMDFA < 90°. The neck-shaft angle **(NSA)** was the angle of intersection between the femoral neck axis and the proximal femoral shaft axis. The normal value of the NSA is 124°-136°. The femoral bowing angle (**FBA**) is the angle formed by **FsFc** and the line of the extended **FdFc** (without bowing, 0°; varus bowing, +; valgus bowing, -) [9]. **aMDFA** was the medial angle formed between the distal femoral anatomical axis and the knee joint line of the femur in the frontal plane. The normal value of the aMDFA = 99° (from 97° to 101°). All angles are shown in Fig 1.

### Influencing factors for femoral mechanical axis varus

**Fig 2:**
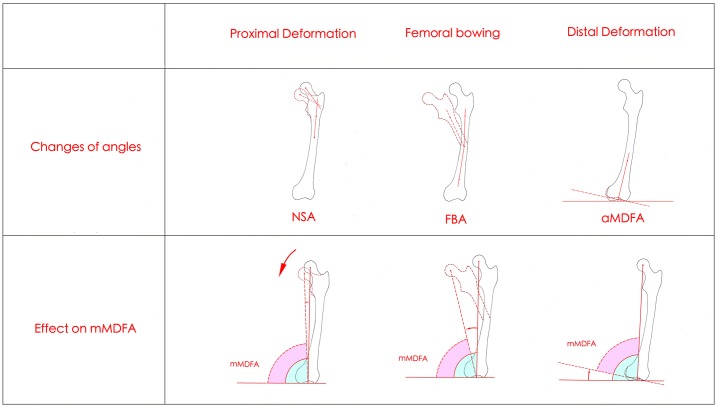
Three morphology changes effect the mMDFA. Proximal deformation (NSA changes): a decreasing NSA leads to a decreasing mMDFA; Femoral bowing (FBA changes): an increasing FBA leads to a decreasing mMDFA. Distal deformation (aMDFA changes): a decreasing aMDFA leads to a decreasing mMDFA.

Femoral mechanical axis varus could theoretically be attributable to three independent geometric changes in the coronal plane: proximal deformation (NSA changes), femoral bowing (FBA changes) and distal deformation (aMDFA changes). Their respective effects on the mMDFA are shown in Fig 2. The different combinations of the three changes lead to a final change in mMDFA.

### Statistical analysis

All data were analyzed using SPSS 19.0 version software for Windows (IBM, Armonk, NY, USA). The repeatability of the measurement method was verified with a consistency test. The Shapiro-Wilk and Kolmogorov-Smirnov tests were used. Histograms and QQ-plots were drawn to determine whether the data were normally distributed. The normality of continuous variables was assessed using the Kolmogorov-Smirnov test. After grouping the data, Kruskal-Wallis and Mann-Whitney U tests were used to test whether there was a significant difference between groups. Correlation analyses were performed using Pearson’s coefficient (parametric data) or Spearman’s coefficient (nonparametric data). Within some variables, partial correlation tests were used. Regression tests were then used for some variables that were associated with each other. Probability values less than 0.05 (two-tailed) were considered indicative of statistical significance.

## Results

### Baseline characteristics

In total, 883 lower extremities of 1538 patients, including 1187 limbs of 684 females and 351 limbs of 119 males, were selected for the study. To test the reliabilities of the radiographic assessments, two orthopedic surgeons performed radiographic measures of the NSA in 30 randomly selected knees twice, with an interval of one month. The measurements were evaluated using the intraclass correlation coefficient (ICC), and all the ICCs were >0.9 (p<0.01) (Table 1). That is, the measurements of intra- and interobserver were reliable.

**Table 1:** Intraclass correlation coefficient(ICC) of measurements.

	NSA 1	NSA 2	ICC	p
Observer A	127.83±4.96	127.70±4.91	0.987	0.000
Observer B	127.97±5.22	128.17±5.13	0.993	0.000
ICC	0.994	0.990		
p	0.000	0.000		

The mean ages (and standard deviation) of females and males were 60.97±8.11 and 60.49±10.18, respectively. The angles for females and males are shown in Table 2.

**Table 2:** Statistical description of variables.

	Female	Male	P
N	1187	351	
Age(year)	60.97±8.11(25~85)	60.49±10.18(17~87)	0.718
HKA(°)	172.71±4.91(151~180)	173.42±4.54(155~180)	0.02
mMDFA(°)	91.30±2.79(80~100)	91.13±2.59(82~98)	0.267
NSA(°)	128.68±6.28(97~149)	127.95±5.64(111~146)	0.032
FBA(°)	2.40±2.63(-8~14)	1.82±2.26(-5~9)	0.001
aMDFA(°)	97.33±2.56(88~107)	97.04±2.24(91~103)	0.032

### Age- and sex-based analysis

The data for females and males were separated into three groups depending on age: <40, 40–60, and >60. Mean angles within the groups showed some differences. As the data were not normally distributed, a nonparametric Kruskal-Wallis test was performed to check the differences among groups, and differences were found to be significant for all variables in females (p<0.05). The Mann-Whitney U test was performed between groups in pairs. Among the <40 and 40–60 groups, there was a significant difference in the NSA and FBA (p<0.01). Between the 40–60 and >60 groups, the mMDFA, FBA, and aMDFA were significantly different (p<0.01). However, in males, differences among age groups had no significance in any of the variables (p>0.05) (Table 3).

**Table 3:** Mean values of groups.

	female(1187)				male(351)			
	mMDFA(°)	NSA(°)	FBA(°)	aMDFA(°)	mMDFA(°)	NSA(°)	FBA(°)	aMDFA(°)
<40	92.25±1.36	134.92±3.85	-0.17±1.40	95.50±2.20	92.75±2.55	130.13±3.68	0.25±1.91	96.50±2.51
40–60	91.50±2.81	128.35±6.37	1.70±2.46	96.99±2.68	91.30±2.42	128.26±6.14	1.92±2.20	97.05±2.22
>60	91.13±2.79	128.84±6.18	3.03±2.61	97.64±2.42	90.91±2.70	127.59±5.24	1.81±2.31	97.06±2.26
P	0.03	<0.001	<0.001	<0.001	0.084	0.216	0.104	0.802

### Correlations between variables

**Fig 3:**
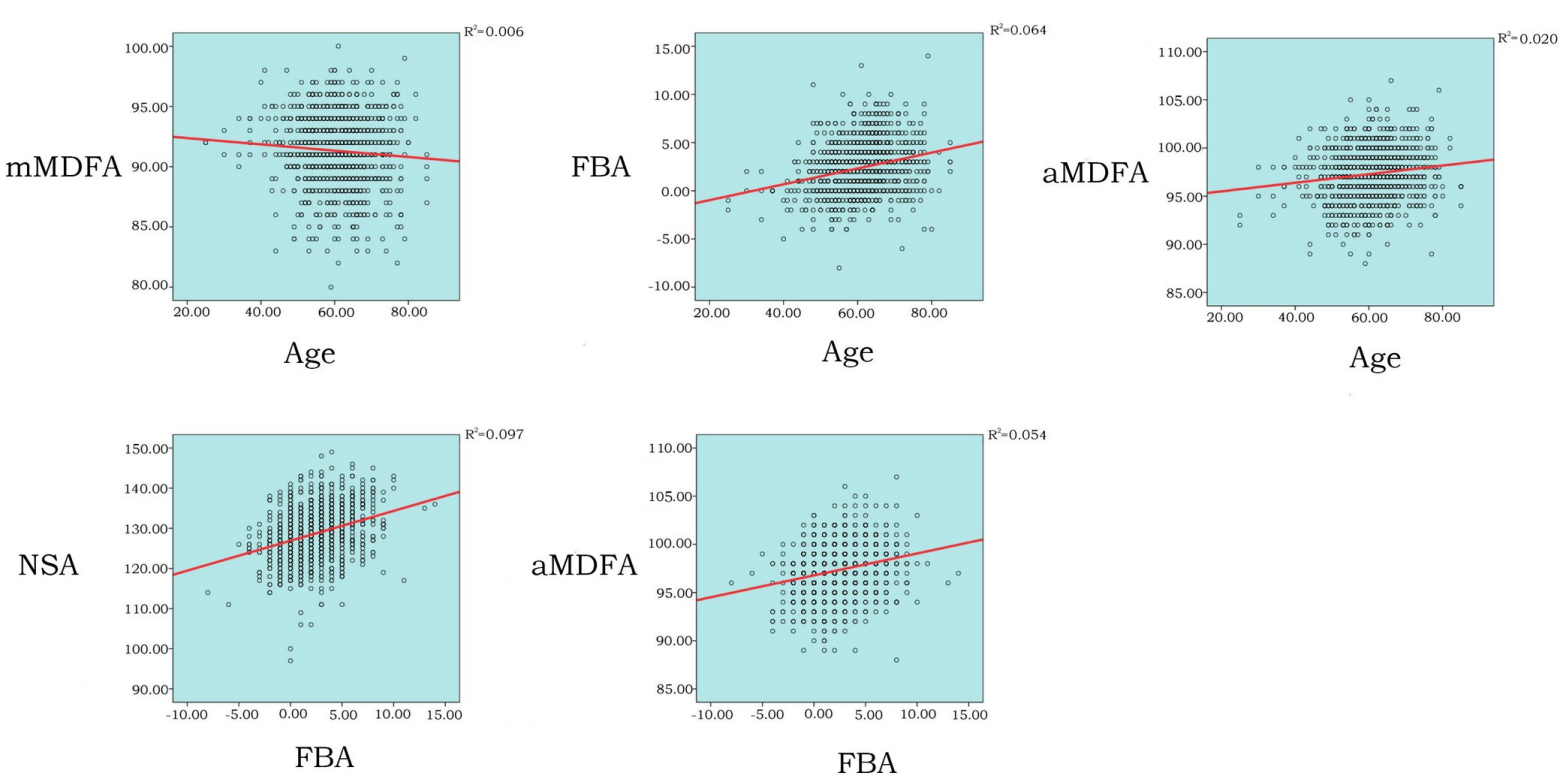
Relations between variables (Female). mMDFA: mechanical medial distal femoral angle; FBA: femoral bowing angle; NSA: neck-shaft angle; aMDFA: anatomic medial distal femoral angle.

In females, as we visualized some relationships between variables by drawing scatterplots; to determine the strength of the relationships, correlation tests were performed (Fig 3). The Pearson correlation test was then used for all measurements because they were classified as continuous variables and the sample size was large. Table 4 shows that there were significant correlations between age and the mMDFA, FBA, and aMDFA (r = -0.076, 0.253, and 0.141, respectively, p<0.01). Significant correlations were found between the FBA and NSA and between the FBA and aMDFA (r = 0.312 and 0.233, respectively, p<0.01). In the partial correlation test between age and the NSA, with the FBA controlled, a negative correlation (r = -0.065, p<0.05) was found. In males, age and all other measurements showed no correlation (Table 5).

**Table 4:** Pearson correlation test between variables (female).

	age	mMDFA	NSA	FBA	aMDFA
age	1	-0.076** 0.009	0.019 0.506	0.253** 0.000	0.141** 0.000
mMDFA		1	0.014 0.618	-0.318** 0.000	0.689** 0.000
NSA			1	0.312** 0.000	0.135** 0.000
FBA				1	0.233** 0.000
aMDFA					1

** represents statistically significant.

**Table 5:** Pearson correlation test between variables (male).

	age	mMDFA	NSA	FBA	aMDFA
age	1	-0.098 0.068	-0.074 0.168	0.041 0.445	0.009 0.865
mMDFA		1	0.198** 0.000	-0.352** 0.000	0.651** 0.000
NSA			1	0.104 0.052	0.084 0.118
FBA				1	0.199** 0.000
aMDFA					1

** represents statistically significant.

### Results of the regression analysis

According to the influencing factors for femoral mechanical axis varus (Fig 2), we hypothesized that in females, the morphological contributors to femoral mechanical axis varus (mMDFA decrease) were the NSA, FBA, and aMDFA. Regression analysis is designed to show the influence of variables on a dependent variable and prove the validity of the results of a correlation analysis. Multiple regression analysis showed that the NSA, FBA and aMDFA together affected 72.2% of the mMDFA (adjusted R square = 0.722); the aMDFA had the most powerful positive influence on the mMDFA (β = 0.803, p<0.01); the FBA had the second largest negative affect on the mMDFA (β = -0.528, p<0.01); and the NSA had a positive and weak influence (β = 0.071, p<0.01) (Table 6). In males, the multiple regression analysis was very similar to that in females (Table 6). That is, the morphological contributors to femoral mechanical axis varus (mMDFA decrease) were the NSA, FBA, and aMDFA for all people.

**Table 6:** Regression test between variables.

	NSA	FBA	aMDFA	Adjusted R Square
mMDFA(femal)	0.071	-0.528	0.803	0.722
mMDFA(male)	0.190	-0.518	0.738	0.698

## Discussion

### Clinical implication of the *mMDFA*

One of the classical components of KOA is knee varus. The majority of previous studies investigating KOA have focused on the proximal tibia. The phenomenon of "nonuniform settlement" suggested that with more pressure in the medial compartment and no bony support, the medial tibial plateau collapses, causing more overload on the medial compartment and leading to a vicious circle that subsequently accelerates the settlement of the medial tibial plateau. "Settlement" could be considered as the start of the vicious circle [3,10]. Age was a significant risk factor for KOA, as it represented the length of weight bearing [11]. Unlike the tibia, the deformation of the femur is easier to neglect. The morphological changes of the involved proximal tibia could be determined by comparison with an ideal one (MPTA = 90°), and the degree of medial tibial plateau collapse could indicate the location and severity of pressure overload. Similarly, supposing that the morphological changes of the femur are due to KOA, the changes should also represent the differences between the involved femur and an ideal one (mMDFA = 90°). The importance of femoral mechanical axis varus has been proven. Cooke et al. [12] first mentioned the femoral component in varus gonarthrosis. Ahmet et al. [4] suggested that the femoral side contributes to the varus alignment as much as the tibial side, after analyzing 315 lower limbs in 164 patients who underwent high tibial osteotomy (HTO) due to varus gonarthrosis. In our study, the largest change of mMDFA fall to 10° of the neutral mechanical axis (mMDFA = 80°); that is, femoral deformity should not be overlooked.

Early stage varus gonarthrosis in middle age is usually successfully treated by correctional osteotomies, which could be considered knee-salvage treatments, instead of TKA, and removing the cause by correcting the knee joint alignment and loading to delay or avoid a second surgery [13]. The current knee-salvage treatment strategies, such as high tibial osteotomy (HTO) [14] and fibular osteotomy [15–17], corrected the alignment of the extremities by changing the anatomical geometry of the distal part of the knee. In contrast, surgeons are always blinded to the deformity of the femur, as there have been few published studies reporting minimally invasive surgery focused on the femur in varus gonarthrosis. J.A.D. van der Woude et al. [18] reported that biplane distal lateral closed-wedge valgus osteotomy of the femur for the treatment of varus deformity of the knee is a valuable procedure when the deformity is localized in the femur. Saragaglia et al. suggested that computer-assisted combined distal femoral and proximal tibial osteotomy in severe genu varum is a reliable, reproducible, and accurate technique [19]. Some studies indicated that remaining mild varus limb alignment leads to better clinical outcomes in TKA for varus osteoarthritis [20,21]. Nevertheless, the mMDFA was the fundamental criterion for femoral orthomorphia. Meanwhile, dynamic deformation was observed in our study. In females, the mMDFA decreased with age. In males, a tendency toward a decreased mMDFA with age was also found, although the result was not significant. These findings suggest that more attention should be paid to the femurs of elderly people, especially females. Given, we highlight the concept of mMDFA over the mechanical lateral distal femoral angle to underscore its unique implications, as the medial distal femur that bore more force was the most likely location of the deformity.

### Three deformations for femoral mechanical axis varus

**Fig 4:**
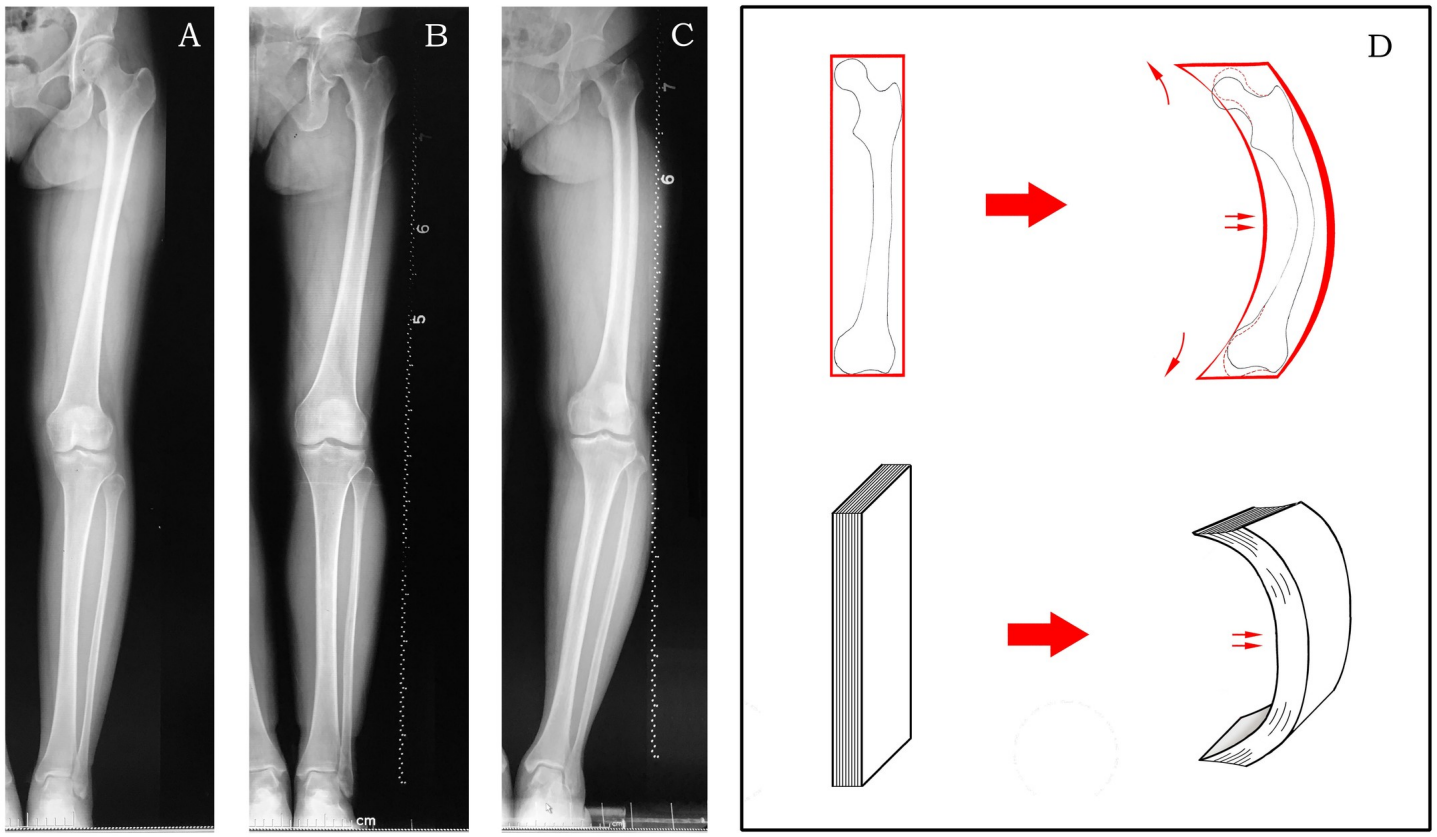
With increasing age, the FBA increased, then the NSA and the aMDFA increased, which finally led to a decreased mMDFA. A. Female, 37 years old, NSA = 133°, FBA = 0°, aMDFA = 96°, mMDFA = 94°. B. Female, 54 years old, NSA = 133°, FBA = 4°, aMDFA = 98°, mMDFA = 90°. C. Female, 63 years old, NSA = 138°, FBA = 7°, aMDFA = 99°, mMDFA = 88°. D. The proximal and distal deformities caused by femur bowing are very similar to the geometric changes in a curling book.

Femur bowing, which could lead to a decreased mMDFA, was a deformation that was easily observed. Yau et al. [22] first reported that the phenomenon of femur bowing was very common in Chinese individuals, with a prevalence of 62%. Femur bowing had a great effect on the distal femoral valgus resection angle (DFVRA) for distal femoral resections in TKA [23], and a fixed 6° DFVRA in these patients could result in unacceptable planning and actual error in limb alignment [24–26]. In this study, we speculated that the cause of female femur bowing could be long-term overloads of the medial compartment, as the results showed that femur bowing was positively associated with age (Table 4). More importantly, we found that femur bowing is not the only deformation responsible for femoral mechanical axis varus, as proximal deformation and distal deformation of the femur could also change the mMDFA (Fig 2). We also found that in females, the FBA had a statistically significant positive correlation with the NSA and the aMDFA (r = 0.312 and 0.233, respectively, p<0.01). That is, the proximal and distal femur were remodeled with an increased FBA. Typical X-ray films from different age groups are shown in Fig 4 (Fig 4A, 4B and 4C). Thus, the deformation was throughout the femur rather than in a local area, which was similar to the geometric changes in a curling book (Fig 4D). Yin et al. [27] indicated that age made an independent contribution to the NSA. Geffrey[28] revealed that a varus NSA is subjected to higher mechanical stress than a normal femoral neck angle. In our study, in the partial correlation test between age and the NSA, while the FBA was controlled, a negative correlation (r = -0.065, p<0.05) was found. We also found that age was positively associated with the aMDFA, indicating that there was a trend toward a decreased NSA and an increased aMDFA during longer-term medial knee joint overloads. Considering the remodeling effect of femur bowing, the final decrease of the mMDFA could be partly compensated. In males, age and all other measurements showed no correlation (Table 4).

### Sex difference

Our results indicate that as KOA progresses, dynamic deformation of the femur could be found in females, while no obvious changes were found in males. Numerous reports have shown that females are at higher risk of arthritis, osteoporosis and autoimmune diseases than males [29–33]. This is probably related to estrogen deficiency and osteoporosis around the time of the menopause, and intensification of deformation accelerates the progression of KOA [34]. It is estimated that 60% of patients who underwent TKA were females [35,36]. Our practice generated 1538 lower extremities, with 1187 knees of females (77.18%) and 351 knees of males (22.82%), all of which points to the same conclusion.

In the absence of large-scale prospective research, identifying the existence of femur varus and its role in the pathogenesis of medial compartment OA are fundamentally limited. The influence of weight and height were not considered. Finally, this study was a component of our research series, and further clinical research will be carried out.

## Conclusions

During medial compartment KOA, dynamic deformation of the femur could be found in females, while no obvious changes were found in males. Femoral mechanical axis varus (mMDFA decrease) was the result of changes in the NSA, FBA and aMDFA. The deformation was throughout the femur rather than in a local area, as femur bowing could lead to corresponding changes in both ends of the femur. We provided the theoretical basis for TKA and knee-salvage treatment. More attention should be paid to aging patients, especially females, in the preoperative protocol for orthomorphia.

## Supporting information

S1 File STROBE statement. (DOCX)

S1 Table The original data of our research about measuring (female). (XLSX)

S2 Table The original data of our research about measuring (male). (XLSX)
